# Mental health promotion in youth sporting clubs: predictors of stakeholder participation

**DOI:** 10.1186/s12889-023-15377-5

**Published:** 2023-03-13

**Authors:** Jasmine M Petersen, Murray Drummond, Sarah Crossman, Sam Elliott, Claire Drummond, Ivanka Prichard

**Affiliations:** 1 College of Education, Psychology and Social Work, Flinders University, GPO Box 2100, Adelaide, South Australia 5001 Australia; 2 SHAPE Research Centre, Flinders University, Adelaide, Australia; 3 Caring Futures Institute, College of Nursing and Health Sciences, Flinders University, Adelaide, Australia

**Keywords:** Sport, Mental health, Young people, Youth sport clubs, Mental health literacy

## Abstract

**Background:**

Young people are disproportionately affected by poor mental health. Youth sport settings hold immense potential to improve the mental health outcomes of this demographic. Efforts to leverage youth sport settings to promote mental health are limited by the lack of knowledge pertaining to engagement with mental health interventions in these settings. Therefore, this study aimed to examine the willingness of youth sporting club stakeholders (e.g., sportspersons, coaches, support staff, parents/guardians) to engage in mental health initiatives conducted by sporting clubs and ascertain possible determinants of engagement.

**Methods:**

This study used an observational cross-sectional design. Participants completed an online survey assessing likelihood of supporting a mental health campaign, mental health literacy (help-seeking, inclusive attitudes), and perceived club support. Perceptions pertaining to the importance of youth mental health and sporting clubs supporting youth mental health were also assessed.

**Results:**

The survey was completed by 275 stakeholders of youth sporting clubs in Australia (M_age_ = 40.2 ± 15.8 years, 60.3% female). The findings indicated that stakeholders were willing to participate in mental health initiatives in youth sport clubs. A linear regression analysis indicated that the significant predictors of stakeholders supporting such initiatives were older age (> 25–50 and > 50 years; β = 0.15, *p* = .033, β = 0.19, *p* = .005, respectively), along with perceived importance of youth mental health (β = 0.24, *p* = .003) and sporting clubs supporting youth mental health (β = 0.22, *p* = .004).

**Conclusion:**

Youth sport settings have the capacity to improve the provision of mental health support among young people. There is a need for tailored approaches to enhance the engagement with, and effectiveness of, mental health resources in sport contexts.

**Supplementary Information:**

The online version contains supplementary material available at 10.1186/s12889-023-15377-5.

## Introduction

Poor mental health is one of the most pervasive issues facing young people. In Australia, for example, 39.6% of young people (16–24 years) are reported to experience a mental health condition (most often anxiety or affective disorders) [1]. Mental health conditions are associated with increased risk of chronic disease and disability [2], in addition to lower academic performance, unemployment, and decreased quality of life (QOL) [3–5]. Concerningly, mental health conditions are also an important risk factor for suicide [6], the leading cause of death among young Australians [7]. Typically, most mental health problems have their onset prior to 24 years of age [8], and alarmingly, these track into later life [9]. This is a critical period to implement innovative approaches to optimise the mental health outcomes of young people.

Harnessing sport settings provides a promising approach to improving the mental health of young people. More specifically, such settings present an important platform to reach and engage this demographic with mental health supports, given evidence indicating that 78.8% of young people in Australia (12–24 years) participate in organised sport [10]. Accordingly, a range of mental health programs have been developed for implementation in sport settings. An emerging body of research suggests that such programs demonstrate the potential to support mental health and well-being [11–13]. A recent systematic review reported positive effects of sport-based interventions (*n* = 28) on mental health (anxiety, stress) and mental health literacy outcomes (mental health knowledge, help-seeking, health referral efficacy) [13]. Notably, however, a meta-analysis of 19 sport-based interventions found mixed evidence regarding the efficacy of such interventions on mental health outcomes (e.g., wellbeing, psychological distress, stigmatising attitudes, mental health literacy) [12]. The authors therefore, concluded that there is considerable scope to improve the effectiveness and quality of mental health interventions implemented in sport settings [12].

Efforts to enhance the engagement with mental health interventions in sport contexts may be key to optimising the effectiveness of such interventions. In a recent evaluation of *Ahead of the Game*, a sports-based mental health and resilience training program, it was found that few adolescent sportspersons (30.0%) participated in all components of the program [14]. Hurley et al. [15] similarly reported challenges pertaining to engagement and reach in the evaluation of a mental health literacy intervention targeted at parents in community sporting clubs. Accordingly, a recent international consensus statement developed to assist effective mental health promotion in sport has recommended that evidence-based guidance is necessary regarding program engagement and implementation, and must acknowledge diversity (e.g., age, gender) [16]. Examining engagement with mental health interventions in sport settings, and more specifically, factors that are linked to such engagement, is critical to advancing this burgeoning field of research.

Sport settings present novel prospects for improving the mental health outcomes of young people. Despite growing efforts to leverage youth sport settings to promote mental health, it is recognised that there is a need to further improve the utility of such approaches. Currently, there is a dearth of knowledge pertaining to engagement with mental health interventions in sport contexts, limiting successful implementation and effectiveness of such interventions. Therefore, this study aimed to examine the willingness of stakeholders (e.g., sportspersons, coaches, support staff, parents/guardians) to engage in mental health initiatives conducted by youth sporting clubs. Additionally, this study ascertained the possible determinants of engagement, and more specifically, the role of individual difference characteristics (e.g., age, gender, mental health attitudes) and environmental factors (club support). This is critical to informing the design and implementation of mental health initiatives in youth sporting contexts that successfully engage key stakeholders, and ultimately, facilitate widespread improvements in the mental health outcomes of young people.

## Method

### Study Design

This study used an observational cross-sectional design. The reporting adheres to the Strengthening the Reporting of Observational Studies in Epidemiology (STROBE) guidelines (see Appendix 1) [17].

### Participants

The sample comprised 275 stakeholders (e.g., sport participant, parent/ guardian of sport participant, president, coach, volunteer; aged ≥ 12 years) of youth sport clubs in Australia. Stakeholders were recruited online via social media (Facebook, Twitter, and LinkedIn) and email contact with sporting organisations (e.g., Sport SA, South Australian National Football League) and the Breakthrough Mental Health Research Foundation. All participants provided informed consent electronically prior to participation, and those aged < 18 years were required to obtain consent from a parent/ guardian.

### Procedure

Ethical approval was granted by the University’s Human Research Ethics Committee (Protocol ID. 4182). Data were collected between August 2021 and July 2022 using an online survey administered via the Qualtrics platform. The survey incorporated the measures outlined below and took approximately 10 min to complete. Participants were provided with the opportunity to enter a raffle to win one of ten AUD50 gift vouchers in recognition of their time commitment.

### Measures

#### Perceptions pertaining to the promotion of mental health in sporting clubs.

Three items using 5-point Likert scales were developed for the present study to examine the perceived importance of youth mental health and sporting clubs supporting youth mental health (1 = *not at all important*, 5 = *extremely important*), and satisfaction with the club’s current level of mental health support provided to youth (1 = *not at all satisfied*, 5 = *extremely satisfied*). All participants were also asked to indicate how likely they would be to support a mental health campaign run by their club on a 7-point Likert scale (1 = *not at all likely*, 7 = *extremely likely*).

#### Mental health literacy.

Three items from the Mental Health Literacy Scale [18] were adapted for applicability to a sport-specific context, and were utilised to assess help-seeking (2-items) and inclusive attitudes (1-item). The items pertaining to help-seeking (e.g., “I am confident that I know where to seek information about mental health” and “I would be comfortable approaching someone at my club about my mental health and wellbeing”) were rated on a 5-point Likert scale (1 = *strongly disagree*, 5 = *strongly agree*). Participants were asked to respond to these items in relation to young people within the sporting club (e.g., youth sport participants responded for themselves, parents/ guardians responded in relation to their child, and all other stakeholders were asked to respond in terms of young people at the club). Inclusive attitudes (as an indicator of stigma) were assessed by asking all participants to rate their willingness to have someone with a mental health issue play at their club (1 = *definitely unwilling*, 5 = *definitely willing*). A higher score on this item indicating more inclusive attitudes (i.e., lower stigma).

#### Club support.

Following Drummond et al. [19], perceived club support was assessed using an adapted version of the Multidimensional Scale of Perceived Social Support [20]. The measure consists of 5 items rated on a 5-point Likert scale from 1 (*strongly disagree*) to 5 (*strongly agree*), and as specified above, the stakeholders responded according to their role within the sporting club. The 5 items were averaged to generate a composite score, with a higher score indicating higher perceived club support. This scale has previously demonstrated good internal consistency (α = 0.86; [19]), and this was similarly shown in the present sample (α = 0.86).

#### Demographic Information.

Stakeholders were asked to report personal characteristics including age (years; categorised as 12–25, > 25–50, > 50), gender, and postcode (to determine Socio-Economic Indicators for Areas (SEIFA) Index of Relative Disadvantage). SEIFA is a measure of socioeconomic economic status derived from the Australian Bureau of Statistics census information that ranks areas in Australia according to relative socioeconomic levels (from *most disadvantaged* [1] to *most advantaged* [10]; [21]). Information pertaining to stakeholders’ sporting club involvement (i.e., main role, sporting code) was also obtained.

### Statistical analysis

Data were analysed using Statistical Package for the Social Sciences version 27 (IBM, Corp). Alpha was set at 0.05. The study variables did not deviate substantially from normality based on skewness, kurtosis, or histogram examination. Therefore, parametric tests were used for all analyses. Descriptive statistics were calculated for all variables. A series of independent *t*-tests and ANOVAs were conducted to assess differences across all outcome variables according to participant characteristics (age, gender). Pearson correlations were used to determine bi-variate relationships between the continuous variables. A linear regression analysis was conducted to identify the predictors of the likelihood of stakeholders supporting a mental health campaign run by a sporting club. The linear regression model incorporated age, gender, SEIFA rank of location, perceived importance of youth mental health and sporting clubs supporting youth mental health, satisfaction with the mental health support provided by clubs, mental health literacy (help-seeking and inclusive attitudes), and perceived club support. All assumptions for linear regression were met (i.e., independence of observations, linearity and homoscedasticity, normality, multicollinearity, and undue influence). A post-hoc power analysis (G*Power) for linear regression indicated that the sample size (*n* = 275) was adequate to detect a medium sized effect (*f*^2^ = 0.15) with 90% power and alpha level of 0.05 [22].

## Results

### Sample

The sample incorporated 275 stakeholders of youth sporting clubs, aged 12 to 78 years (M_age_ = 40.2 ± 15.8 years, 60.3% female). They reported diverse roles in their respective clubs including parent/guardian of a sportsperson (36.0%), current youth player (15.0%), coach (14.0%), committee member (10.2%), general volunteer (9.4%), CEO or president (5.1%), team manager (2.5%), and umpire or referee (2.2%). The main sporting codes participants were involved with included hockey (21.8%), Australian Rules Football (17.1%), baseball (8.4%), cricket (6.5%), netball (5.1%), and soccer (4.7%).

As shown in Table 1, there were several significant differences on the variables pertaining to mental health promotion in sporting clubs according to age and gender. Specifically, youth stakeholders (12–25 years; *F*(2, 270) = 4.9, *p =* .007) and males (*t*(270) = 2.5, *p* = .014) reported a significantly lower likelihood of supporting a mental health campaign conducted by sporting clubs. Males (relative to females) perceived greater club support for youth mental health (*t*(237) = 2.7, *p* = .007), however, they also rated youth mental health and sporting clubs supporting youth mental health as less important (*t*(270) = 3.6, *p* < .001; *t*(270) = 3.0, *p* = .003, respectively).

**Table 1:** Means (and standard deviations) across variables related to mental health promotion in sporting clubs by sample characteristics

	Likelihood of supporting mental health campaign run by club	Importance of youth mental health	Importance of sporting clubs supporting youth mental health	Satisfaction with mental health support provided by sporting club	Mental health literacy; help-seeking	Mental health literacy; inclusive attitudes	Club Support
Overall (*n* = 275)^a^	6.1 (1.2)	4.7 (0.5)	4.5 (0.7)	3.2 (1.1)	3.0 (0.8)	4.6 (0.7)	3.7 (0.8)
Age (years)							
12–25 (*n* = 70)	5.7 (1.3)^b^	4.6 (0.6)	4.4 (0.7)	3.1 (1.2)	3.1 (0.8)	4.6 (0.8)	3.7 (0.8)
> 25–50 *(n* = 125)	6.2 (1.1)^c^	4.8 (0.5)	4.5 (0.7)	3.2 (1.1)	3.0 (0.9)	4.6 (0.7)	3.7 (0.8)
> 50 *(n* = 78)	6.3 (1.0)^c^	4.7 (0.5)	4.5 (0.6)	3.3 (1.1)	3.2 (0.8)	4.6 (0.7)	3.7 (0.7)
Gender							
Male (*n* = 106)	5.9 (1.3)^b^	4.6 (0.6)^b^	4.3 (0.8)^b^	3.3 (1.1)	3.2 (0.9)	4.7 (0.7)	3.9 (0.7)^b^
Female (*n* = 166)	6.2 (1.0)^c^	4.8 (0.4)^c^	4.6 (0.6)^c^	3.1 (1.1)	3.0 (0.8)	4.6 (0.7)	3.6 (0.8)^c^

*Notes*

^a^Total number in each row may not equate to 275 due to missing responses.

^b,c^Means with different subscripts demotes significant differences (*p* < .05), i.e., group *a* is significantly different from group(s) *b.*

### Associations between variables related to mental health promotion in sporting clubs

Correlations between variables pertaining to the promotion of mental health in sporting clubs are presented in Table 2. The likelihood of sporting club stakeholders supporting a mental health initiative run by the club was significantly positively associated with both perceived importance of youth mental health and sporting clubs supporting youth mental health, in addition to mental health literacy (inclusive attitudes). Perceived importance of youth mental health and sporting clubs supporting youth mental health were positively associated with mental health literacy (inclusive attitudes) and SEIFA rank of location. Satisfaction with the mental health support provided by sporting clubs was significantly positively associated with mental health literacy (help-seeking) and perceived club support.

**Table 2:** Correlations between variables pertaining to mental health promotion in sporting clubs

	1	2	3	4	5	6	7
1. Likelihood of supporting mental health initiative run by club	1						
2. Importance of youth mental health	0.44**	1					
3. Importance of sporting clubs supporting youth mental heath	0.42**	0.63*	1				
4. Satisfaction with mental health support provided by sporting clubs	0.004	-0.10	0.09	1			
5. Mental health literacy; help-seeking	-0.01	-0.11	-0.06	0.40**	1		
6. Mental health literacy; inclusive attitudes	0.22*	0.32**	0.21**	-0.11	0.007	1	
7. Club support	0.11	0.02	0.12	0.52**	0.50**	0.10	1
8. SEIFA rank of location	0.10	0.16**	0.13*	0.06	-0.04	0.04	0.05

*Note*. *p < .05, **p < .01

### Regression Analyses: Determining overall predictors of the likelihood of stakeholders supporting mental health initiatives conducted by sporting clubs

Results of the multiple regression analysis are presented in Table 3. The regression model accounted for 27.5% of the variance in likelihood of sporting club stakeholders supporting mental health initiatives conducted by such clubs (*R*^2^ = 0.275) and was significant, *F*(10, 228) = 8.6, *p* < .001. The significant positive predictors of supporting mental health initiatives conducted by clubs were older age (> 25 years) and perceived importance of youth mental health and sporting clubs supporting youth mental health.

**Table 3:** Linear regression examining predictors of likelihood of supporting mental health initiatives conducted by sporting clubs

	β	*p*-value	95% CI
Age (years) (12–25 is ref)			
> 25–50	0.15	**0.033**	[0.01,0.29]
> 50	0.19	**0.005**	[0.06,0.33]
Gender			
Female (vs. Male)	0.09	0.154	[-0.03,0.21]
SEIFA rank of location	0.02	0.728	[-0.09,0.13]
Importance of youth mental health	0.24	**0.003**	[0.08,0.40]
Importance of sporting clubs supporting youth mental heath	0.22	**0.004**	[0.07,0.36]
Satisfaction with mental health support provided by sporting clubs	-0.05	0.510	[-0.18,0.09]
Mental health literacy; help-seeking	-0.02	0.979	[-0.13,0.13]
Mental health literacy; inclusive attitudes	0.10	0.148	[-0.03,0.21]
Club support	0.11	0.155	[-0.04,0.25]

*Note*. Bold indicates significance at *p* < .05

## Discussion

The present study aimed to examine the willingness of youth sporting club stakeholders to support mental health initiatives conducted by sporting clubs. This included determining the key predictors of stakeholders’ willingness to support these initiatives. The findings indicated that stakeholders are likely to participate in mental health initiatives in youth sport clubs. Additionally, age and perceptions pertaining to the promotion of mental health in sporting clubs emerged as important predictors of willingness to participate in such initiatives.

Overall, the findings showed that sporting club stakeholders (e.g., players, parent/ guardians of sportspersons, coaches) are willing to engage in mental health initiatives conducted by youth sporting clubs. This provides an important extension to previous research suggesting that various stakeholder samples (adolescent sportspersons, parents of youth, and coaches) perceive organised sport as a valuable setting to promote mental health [23–25]. The present study reinforces the growing consensus that sport settings hold great potential to improve the provision of mental health support [14, 26–28]. Increasing the utilisation of such settings for mental health promotion is critical, particularly given evidence suggesting that few sport organisations in Australia (11.3%) have implemented initiatives to address mental health [29]. Future research could usefully ascertain stakeholders’ preferences pertaining to mental health promotion in sport settings to inform the implementation of initiatives that are acceptable, feasible and broadly scalable.

This study also provides a novel insight into the key factors that impact the engagement of sporting club stakeholders with mental health initiatives in sport settings; this is important to enhancing the design and implementation of these initiatives. The regression analysis showed that age is a significant positive predictor of willingness to support mental health initiatives conducted in sport clubs. That is, older stakeholders (> 25 years) indicated a greater likelihood of supporting such initiatives. This is noteworthy as these stakeholders are more likely to hold positions of leadership in sport settings (e.g., club committee), and thus, may have the capacity to drive the successful implementation and adoption of mental health initiatives. On the other hand, our findings suggest that younger stakeholders (aged 12–25 years) are less likely to participate in mental health initiatives in sporting clubs. This is concerning given that young people have the highest reported prevalence of mental health conditions, in Australia for example, 39.6% of those aged 16–24 years are reported to experience such conditions [1]. Existing research suggests that among young people, there are several barriers to engagement with mental health supports including perceived stigma, negative perceptions towards support services, and limited mental health literacy [30, 31]. As such, targeting these barriers could be key to engaging this demographic in mental health initiatives in sport settings.

An important consideration in addressing barriers to engagement in health interventions, in addition to enhancing the effectiveness of such interventions, is the utilisation of behaviour change theory [32]. Few mental health interventions developed for sporting contexts are shown to be underpinned by behaviour change theory [13]; a key limitation to advancement of this field. Efforts to support the mental health of young people in sport contexts could be enhanced by utilising behaviour change theory such as the popular Capability, Opportunity and Motivation (COM-B) framework [33]. The COM-B framework, proposes that capability (physical and psychological; e.g., mental health knowledge), opportunity (social and physical influences; e.g., supportive environment) and motivation (e.g., stigmatising attitudes) interact to influence behaviour (e.g., help-seeking). The framework also provides intervention strategies (e.g., education, persuasion, incentivisation, modelling) targeted at facilitating capability, opportunity, and motivation that may be beneficial to improving the design and implementation of mental health strategies in sport contexts for young people.

The findings also indicate that perceptions pertaining to the promotion of mental health (importance of youth mental health and sporting clubs supporting youth mental health) predicted the willingness of stakeholders to support mental health initiatives conducted by sporting clubs. This fits with existing evidence suggesting that attitudinal barriers (e.g., low perceived importance or disinterest in mental health promotion) are a key challenge in the implementation of mental health initiatives in sporting contexts [15, 34, 35]. Attitudinal barriers are suggested to stem from poor mental health literacy, along with societal and self-stigma [36]. As such, efforts to improve mental health knowledge, beliefs and awareness, may be critical to facilitating stakeholders’ investment in, and commitment to, establishing sport settings as sites that prioritise mental health. Moreover, Walton et al. [37] recommend that sporting clubs appoint mental health ‘champions’, referring to stakeholders tasked with calling attention to and advocating for the importance of mental health promotion in clubs. ‘Champions’ may usefully provide both visible and accessible mental health leadership and play an important role in addressing attitudinal barriers; critical to driving participation in mental health initiatives conducted by sporting clubs.

Perceived club support did not emerge as a predictor of willingness to support mental health initiatives implemented by sporting clubs. This contrasts with findings from a recent formative evaluation of a sports-based mental health program citing that the social architecture (e.g., strong social networks) of a sporting club is key to successful implementation of such programs [34]. It is possible that social support plays an important role in stakeholder’s actual (rather than intended) engagement in mental health initiatives in sporting clubs. Establishing psychologically safe sport settings (i.e., environments free from psychological harm) known to foster connectedness, belonging and enhanced support, is seemingly pertinent to effectively promoting mental health in such settings. Sporting club stakeholders play an important role in shaping sport settings into safe spaces. For example, coaches (or club leaders) are encouraged to develop and foster positive interpersonal relationships, act as positive role models and demonstrate inclusive leadership to establish safe and supportive sporting environments [38]. This speaks to the growing recognition that multilevel interventions targeting various stakeholders (e.g., sports persons, coaches, parents) are necessary to the promotion of mental health in sport settings [39].

The findings further indicate that mental health literacy (help-seeking or inclusive attitudes) did not predict willingness to engage in mental health initiatives conducted by sporting clubs. Although, a positive correlation was established between inclusive attitudes (i.e., lower stigma) and likelihood of supporting initiatives in sporting contexts, suggesting that such attitudes may nevertheless play an important role. Similarly, SEIFA rank of location did not emerge as a predictor, even though it was positively associated with perceptions pertaining to the importance of youth mental health and sporting clubs supporting mental health; both shown to predict willingness to support mental health initiatives in sport settings. Evidence suggests that individuals from a lower socio-economic background experience an increased prevalence of mental health concerns [40], are less likely to utilise mental health services [41], and exhibit poorer mental health literacy [42]. Consideration should, therefore, be given to demographic factors (e.g., socio-economic status of population) in the design and implementation of mental health interventions in sporting contexts.

The present study has important implications for the promotion of mental health in youth sporting contexts. The findings indicate that youth sporting clubs should be harnessed to improve the provision of mental health support. They also suggest that strategies are necessary to encourage young people to participate in mental health initiatives in sporting clubs. This is particularly important given that mental health concerns most commonly emerge during youth [8], and so engaging this subpopulation in early intervention or prevention efforts is critical. Taken together the findings indicate that a one-size-fits all approach is not appropriate when leveraging sport contexts to support mental health. Specifically, factors including demographic attributes (e.g., age, socio-economic status), mental health attitudes, and the sporting environment (e.g., psychological safety) should be considered in design and/ or implementation of such initiatives. As such, a suite of mental health resources or “tool kit” as proposed by Walton et al. [37] that it sensitive and adaptable to the varied and unique nature of sporting contexts, may pave the way moving forward.

As with all studies there are some limitations that should be acknowledged. More specifically, the cross-sectional nature of this study precludes conclusions of causality. The convenience sampling approach that was adopted may be conducive to self-selection bias, and this may limit the generalizability of the findings. In addition, the assessment of stakeholders’ intentions to participate in mental health initiatives in sporting clubs may not translate into behavioral engagement in such initiatives [43]. Future evaluations of mental health interventions in sport settings should assess outcomes related to both implementation and effectiveness.

## Conclusion

In conclusion, the present study provides a novel contribution to the emerging body of evidence in relation to youth sport contexts as sites for mental health promotion. The findings suggest that youth sport settings hold immense potential to improve the provision of mental health support among young people. Various factors (e.g., age, mental health attitudes) should be considered in the design and implementation of mental health resources in sport contexts. This speaks to the importance of moving away from a one-size-fits-all approach and instead developing tailored approaches that may enhance the engagement with, and effectiveness of, mental health resources in sport contexts. The promotion of mental health in youth sport settings may be fundamental to improving the mental health outcomes of young people, and should therefore, be considered a priority for future research.

## Electronic supplementary material

Below is the link to the electronic supplementary material.

Supplementary Material 1

## Data Availability

The dataset used during the current study is available from the corresponding author on reasonable request.
